# Method for Mitigating Stray Current Corrosion in Buried Pipelines Using Calcareous Deposits

**DOI:** 10.3390/ma14247905

**Published:** 2021-12-20

**Authors:** Sin-Jae Kang, Min-Sung Hong, Jung-Gu Kim

**Affiliations:** 1 School of Advanced Materials Science and Engineering, Sungkyunkwan University (SKKU), Suwon 16419, Korea; paul7751@hanmail.net; 2 Department of Nuclear Engineering, University of California at Berkeley, Berkeley, CA 94720, USA; mshong@berkeley.edu

**Keywords:** stray current corrosion, pipeline, calcareous deposit, corrosion mitigation, cathodic protection

## Abstract

Stray current corrosion in buried pipelines can cause serious material damage in a short period of time. However, the available methods for mitigating stray current corrosion are still insufficient. In this study, as a countermeasure against stray current corrosion, calcareous depositions were applied to reduce the total amount of current flowing into pipelines and to prevent corrosion. This study examined the reduction of stray current corrosion via the formation of calcareous deposit layers, composed of Ca, Mg, and mixed Ca and Mg, at the current inflow area. To verify the deposited layers, scanning electron microscopy (SEM), energy dispersive X-ray spectroscopy (EDS), and X-ray diffraction (XRD) were performed. The electrochemical tests revealed that all three types of calcareous deposits were able to effectively act as current barriers, and that they decreased the inflow current at the cathodic site. Among the deposits, the CaCO_3_ layer mitigated the stray current most effectively, as it was not affected by Mg(OH)_2_, which interferes with the growth of CaCO_3_. The calcium-based layer was very thick and dense, and it effectively blocked the inflowing stray current, compared with the other layers.

## 1. Introduction

Stray current corrosion, which is a drastic corrosion phenomenon due to external current sources, can cause serious damage to buried pipelines in a short period of time. With the increasing number of buildings, facilities, and subways that use high voltages in modern society, the amount of stray current is also increasing, inducing more stray current corrosion in buried pipelines [1,2,3,4,5,6]. In particular, stray current from subways, power towers, and high-voltage facilities flows into buried pipelines that have lower resistances than soil. The area into which the stray current flows is negatively charged, resulting in anticorrosion, while the area out of which it flows is positively charged, resulting in corrosion [1,7,8]. The inflow and outflow of the stray current occur at random areas throughout pipelines, making it difficult to detect, and difficult to prevent the associated corrosion [9,10,11]. In addition, most pipelines are usually installed in urban areas, where stray current can be introduced to the pipelines, causing both economic and human-related losses [12,13]. In the UK, GBP 500 million is spent annually on infrastructure restoration and repair due to stray current corrosion [14]. To solve this issue, drainage systems and electrical shields have been applied to buried pipelines to prevent corrosion [1,15,16,17]. However, they are expensive and cannot be applied to all pipelines. Since it is hard to predict where stray current corrosion will occur, it is difficult to ensure that all pipelines are protected.

To overcome these issues, we applied calcareous deposits for protection against stray current corrosion. Generally, calcareous deposits are a type of combined deposit based on calcium (Ca) and magnesium (Mg), and they are generated under cathodic protection in seawater. Generally, calcareous deposits act as electrical barriers, and they therefore have the potential to be an excellent solution with regard to mitigating stray current corrosion. The cathodic sites of pipelines, where the inflow current is introduced, have the same conditions when cathodically protected. Therefore, the supply of Ca and Mg can generate a calcareous deposit in a soil environment [18,19,20,21,22].

In this study, potentiostatic polarization tests were performed to form calcareous deposits with different compositions [23]. After forming the calcareous deposits, the surface morphology was analyzed using scanning electron microscopy (SEM), energy dispersive X-ray spectroscopy (EDS), and X-ray diffraction (XRD). Electrochemical impedance spectroscopy (EIS) experiments were also performed after each potentiostatic polarization test. Finally, potentiostatic acceleration tests were undertaken to verify the effects of the calcareous deposits on the stray current corrosion, and to determine the most effective composition for protection against it.

## 2. Materials and Methods

### 2.1. Specimen and Solution Preparation

As shown in Table 1, the specimens used in this study were made of SPW-400 (low-carbon steel), which is the most common material for pipelines.

The SPW-400 was cut into square sections (10 mm × 10 mm × 2 mm), which were used as the working electrodes (WE). The specimens were ground with SiC paper (600-grit), after which they were cleaned with deionized water, and then dried with N_2_ gas. Table 2 lists the chemical composition of the synthetic soil solution used in the experiments. For the formation of the calcareous deposits using potentiostatic polarization tests, Ca and Mg, which are the main components of these deposits, were added to the synthetic soil solution, separately and together. The elements were based on the following chemicals: Mg(OH)_2_ (Mg: 1000 ppm), CaCO_3_ (Ca: 1000 ppm), and Ca and Mg (500 ppm each).

### 2.2. Formation of Calcareous Deposits

A potentiostatic polarization test using a three-electrode system was performed to form the calcareous deposits. The specimens were connected to a WE, a carbon rod was used as the counter electrode (CE), and a saturated calomel electrode (SCE) was used as the reference electrode (RE). The area of each test specimen exposed to electrolytes was 1 cm^2^. The open-circuit potential (OCP) was established within 30 min, after which the electrochemical tests were performed. Potentiostatic polarization tests were undertaken to form the calcareous depositions. The tests were performed at −1.0 V_SCE_ to put the specimens in the cathodic state. The current was inflowed for over 30 h at room temperature (25 °C), while the solution was rotated at 350 rpm.

### 2.3. Surface Analyses

The surface analysis of the experimental specimens was performed after the potentiostatic polarization tests. The morphology and the cross-sectional images of the calcareous deposits were observed using SEM and SEM/EDS (JSM-7900F, JEOL Ltd., Tokyo, Japan) to verify the type of calcareous deposit on the specimen. XRD (Dmax-2500V/PC, Rigaku, Tokyo, Japan) measurements were also performed on the calcareous deposits to verify their types. The XRD analysis was conducted using Cu Kα radiation (λ = 1.54056 Å), in a 2θ range of 0–60°, at a scan rate of 0.02.

### 2.4. Electrochemical Test

**Figure 1:**
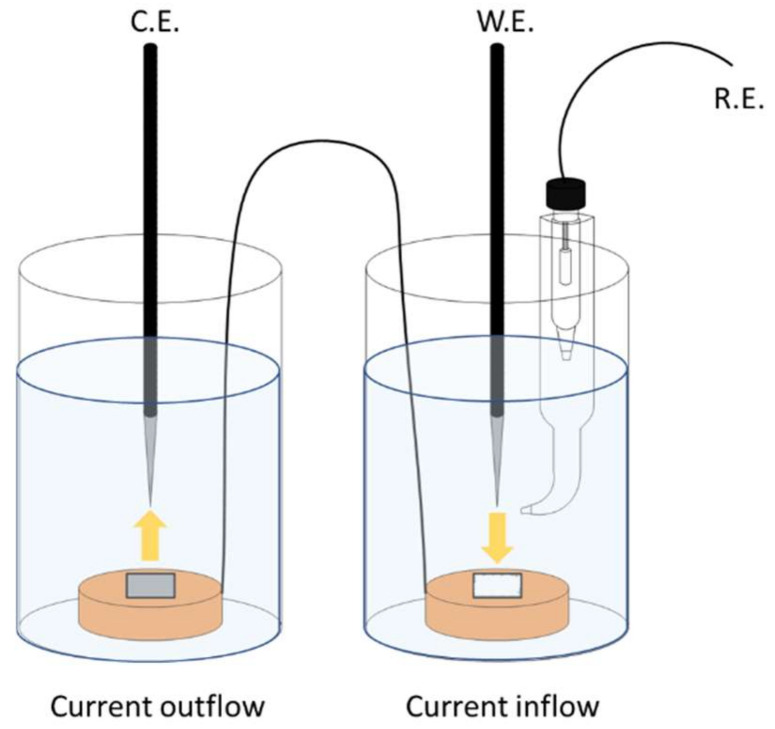
Schematic of stray current simulation cell.

EIS measurements were performed in a frequency range of 100 kHz–10 mHz, with a 10-mV amplitude. The impedance plots were interpreted on the basis of an equivalent circuit, using a fitting procedure performed by ZsimpWin software (ZsimpWin 3.20, Echem Software, Warminster, PA, USA). Stray current corrosion tests were performed in a stray current simulation cell, as shown in Figure 1. The 304 stainless steel rods used as the CE were enclosed with insulating tape to reduce the current dispersion, and the SCE was used as the RE. The specimens used for the inflow part of the current and those used for the outflow part of the current were electrically connected to each other. The tests were conducted in a synthetic soil solution, and 3.5 V_SCE_ was applied for 100 h at room temperature (25 °C). The specimens for the outflow part of the current were weighed and recorded before the potentiostatic acceleration tests. After the tests, the specimens were cleaned, rinsed, and reweighed. All electrochemical tests were performed using a VSP-300 model potentiostat (Biologic SAS, Seyssinet-Pariset, France).

## 3. Results

### 3.1. Formation of Calcareous Deposits

**Figure 2:**
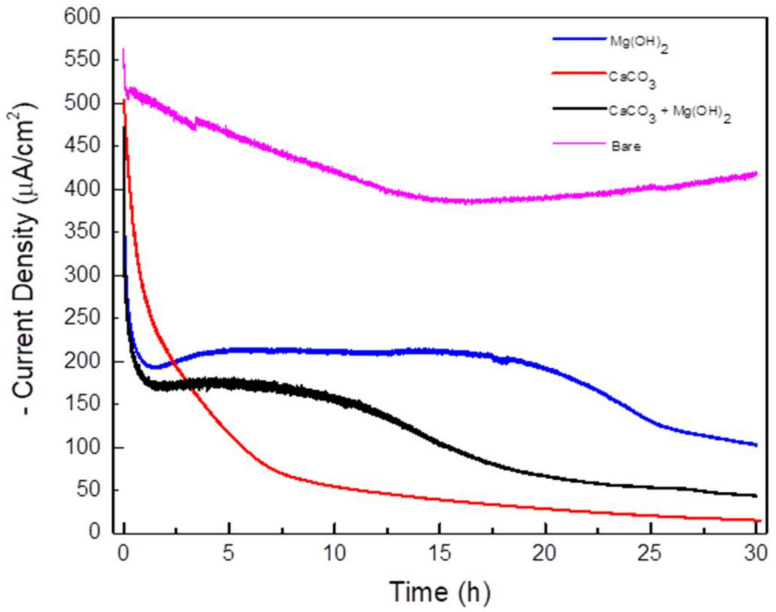
Current density vs. time curves during potentiostatic test (applied voltage: −1.0 V_SCE_; solutions: synthetic soil solution with Mg(OH)_2_ CaCO_3_ and Mg(OH)_2_, CaCO_3_; testing time: 30 h).

The potentiostatic tests were performed at −1.0 V_SCE_ for 30 h to form three kinds of calcareous deposits on the carbon steel. Figure 2 shows the potentiostatic test results over the 30-h period. The current density decreased with time for all solution types. This can be explained by the electrochemical reactions on the surfaces of the cathodic site. When the specimens were negatively charged in the solution containing both Ca and Mg, the dissolved oxygens were converted into OH^−^ ions, leading to an increase in the pH on the surface. Because of the increasing number of OH^−^ ions, Mg ions reacted with them, forming an Mg(OH)_2_ deposition on the metal surface. In addition, the increase in OH^−^ ions affected the carbonate equilibrium at the metal surface. Thus, a CaCO_3_ layer was deposited on the metal surface. These processes can be described by the following reactions [21,22,23,24,25]:

O_2_ + 2H_2_O + 4e^−^ → 4OH^−^ (1)

Mg^2+^ + 2OH^−^ → Mg(OH)_2_ (s) (2)

OH^−^ + HCO_3_^−^ → CO_3_^2^^−^ + H_2_O (3)

Ca^2+^ + CO_3_^2−^ → CaCO_3_ (s) (4)

These calcareous deposits decreased the O_2_ diffusion to the metal surface as a physical and electrical coating layer, and hindered the oxygen reduction reaction [23,26]. Therefore, the current density was decreased because of the formation of calcareous deposits on the metal surface, as shown in Figure 2. However, in the case of the solution with only Mg, the Mg(OH)_2_ layer was porous and gel-like rather than solid. It offered a relatively lower protective property at the surface of the metal compared with the other layers, and it did not significantly decrease the current inflow to the metal [27]. Therefore, the specimen that deposited only Mg(OH)_2_ had the highest current density. In contrast, the CaCO_3_ layer has the property of forming a solid and dense layer. Because the CaCO_3_ layer with these properties grew without any interference, the current density decreased rapidly to the lowest current density value measured in this study [28]. The specimen that deposited both CaCO_3_ and Mg(OH)_2_ had a current density higher than that of the specimen with only CaCO_3_, and a current density lower than that of the specimen with only Mg(OH)_2_. This is because the Mg(OH)_2_ hindered the growth of the CaCO_3_, meaning that the formed CaCO_3_ layer was thin and unstable [28,29].

### 3.2. Surface Analysis

**Figure 3:**
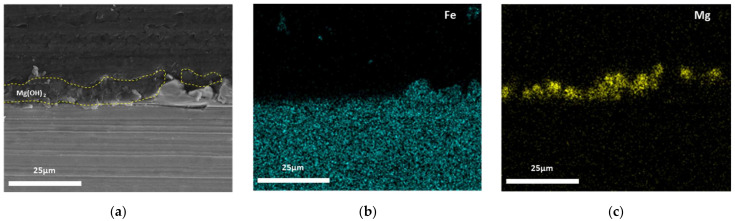
The cross-sectional SEM images and EDS mapping results of Mg(OH)_2_: (**a**) cross-sectional image; (**b**) Fe (EDS mapping); and (**c**) Mg (EDS mapping).

**Figure 4:**
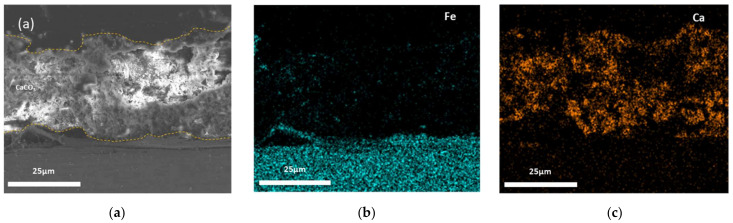
The cross-sectional SEM images and EDS mapping results of CaCO_3_: (**a**) cross-sectional image; (**b**) Fe (EDS mapping); and (**c**) Ca (EDS mapping).

**Figure 5:**
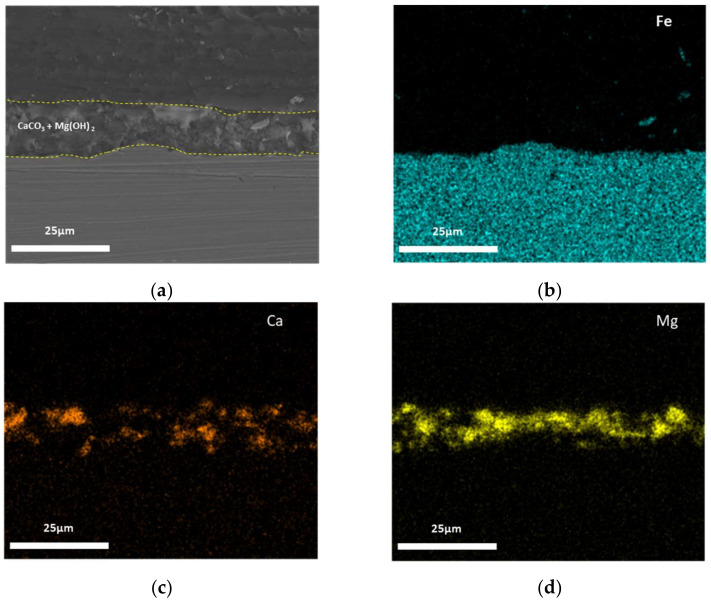
The cross-sectional SEM images and EDS mapping results of CaCO_3_ + Mg(OH)_2_: (**a**) cross-sectional image; (**b**) Fe (EDS mapping); (**c**) Ca (EDS mapping); and (**d**) Mg (EDS mapping).

The cross-sectional SEM images and EDS mapping results of the calcareous deposits after the 30-h potentiostatic polarization tests are shown in Figure 3, Figure 4 and Figure 5. Figure 3 shows this information for the calcareous deposit based only on Mg, revealing a thin Mg(OH)_2_ layer deposited on the carbon steel. Figure 4 shows this information for the deposit based only on Ca, revealing a relatively thicker CaCO_3_ layer deposited on the carbon steel than the other deposit layers. Figure 5 shows the SEM image and EDS mapping results of the mixed CaCO_3_ and Mg(OH)_2_ deposit. This calcareous deposit layer included both Ca and Mg, and can therefore be regarded as a combined CaCO_3_ and Mg(OH)_2_ layer. In addition, it was confirmed that the Mg(OH)_2_, which hindered the growth of the CaCO_3_, resulted in a thinner CaCO_3_ layer compared with the specimen containing only CaCO_3_ [28,29].

**Figure 6:**
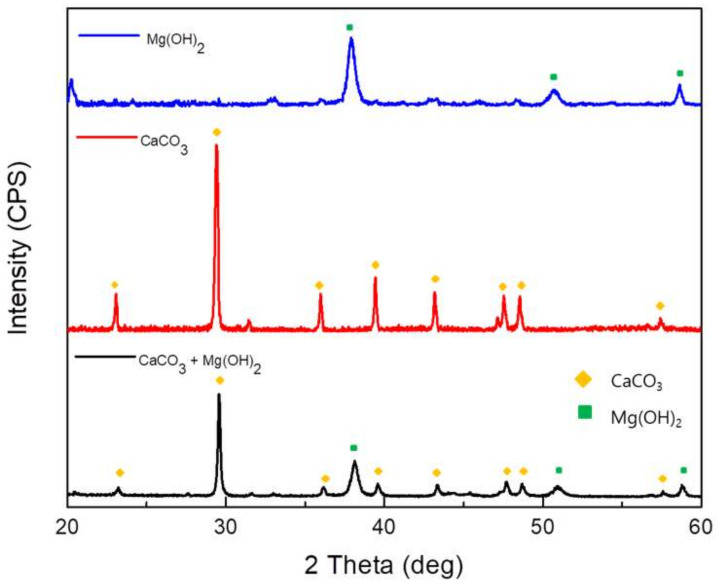
XRD results for the 3 types of calcareous deposits after 30 h of potentiostatic polarization.

Figure 6 shows the XRD patterns used to verify the three types of calcareous deposits on the surfaces of the specimens. It was confirmed that the calcareous deposit layer from the Mg(OH)_2_-added soil solution was Mg(OH)_2_. In addition, the calcareous deposit layer from the CaCO_3_-added soil solution was CaCO_3_. Finally, the calcareous deposit layer from the CaCO_3_ and Mg(OH)_2_-added soil solution consisted of CaCO_3_ and Mg(OH)_2_. When the calcareous deposition layer is formed in a solution to which CaCO_3_ and Mg(OH)_2_ are added, not only CaCO_3_ and Mg(OH)_2_ are formed, but (Ca,Mg)CO_3_ (JCPDS 43-0697), which is similar to the peaks of CaCO_3_ (JCPDS 05-0586), is also formed as the product. The width of the peaks of the calcareous deposit layer from the CaCO_3_ and Mg(OH)_2_-added soil solution are greater than that of the peaks in the other two patterns because of the overlapping XRD peaks of the (Ca,Mg)CO_3_.

### 3.3. Electrochemical Impedance Spectroscopy

**Figure 7:**
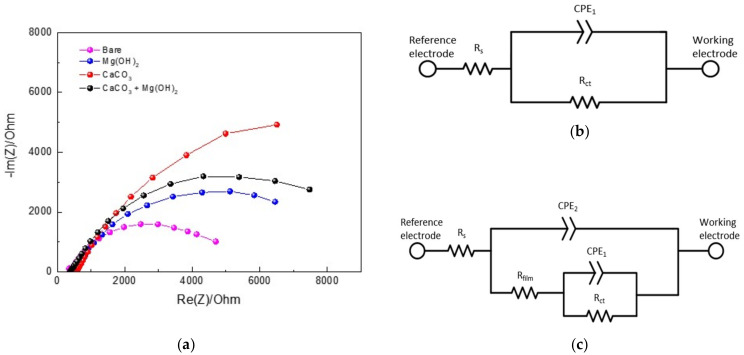
(**a**) Result of EIS test according to the types of calcareous deposits. (**b**) Equivalent circuit diagram of bare carbon steel. (**c**) Equivalent circuit diagram of carbon steel covered by calcareous deposits.

After the 30-h potentiostatic polarization tests, EIS measurements were performed. The Nyquist plots of the data from the electrodes giving different types of calcareous deposits are shown in Figure 7a. The Nyquist plots consist of a capacitive semicircle at a high frequency. Figure 7b presents the equivalent electrical circuit for bare steel, where R_s_ is the solution resistance, R_ct_ is the charge transfer resistance, and CPE_1_ is the double-layer capacitance formed by the electrical double layer that exists at the interface between the electrolyte and electrode [26,30,31]. Figure 7c shows the equivalent electrical circuit that describes the formation of porous calcareous deposits on the surface of the steel. In Figure 7c, R_s_ is the solution resistance; CPE_2_ is the dielectric nature of the calcareous deposits, which is associated with the thickness of the calcareous deposit layer; R_film_ is the pore resistance; CPE_1_ is the capacitance generated by the metal dissolution reaction and by the electric double layer at the solution/metal interface; and R_ct_ is the charge transfer resistance caused by the metal dissolution reaction [25]. Here, a CPE is used instead of a capacitor to compensate for the nonhomogeneity of the system frequency. The impedance of a CPE is described by the following equation:

Z_CPE_ = *A*^−1^(j*ω*)^−n^ (5)

where A^−1^ is the proportionality coefficient (with units, Ω^−1^ s^n^ cm^−2^); ω is the angular frequency (rad s^−1^); j^2^ = −1 is an imaginary number; and n is an empirical exponent (0 ≤ n ≤ 1) that measures the deviation from the ideal capacitive behavior [32,33,34].

**Figure 8:**
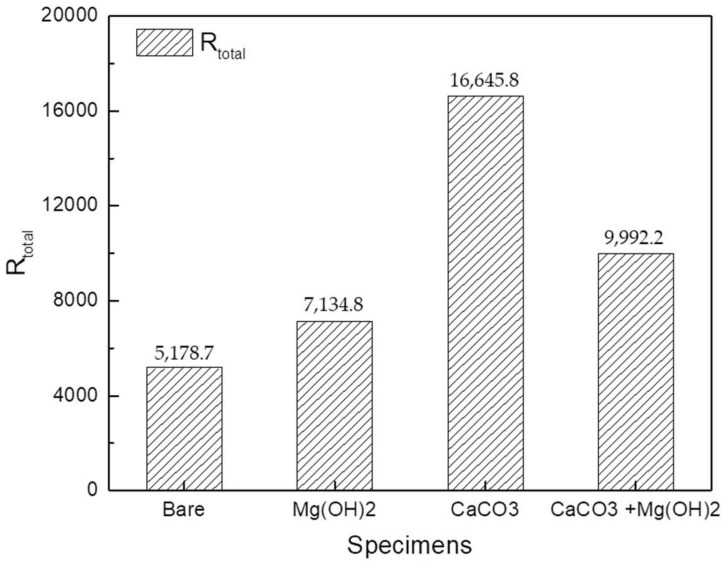
Comparison of total resistance on the bare and 3 types of calcareous deposited specimens.

The results of the EIS fitting using the ZSimpWin software are shown in Table 3. The R_film_ values were the largest in the CaCO_3_ layer, followed by the CaCO_3_ and Mg(OH)_2_ mixed layer, and then the Mg(OH)_2_ layer. Similar to the results for the R_film_ value, CaCO_3_ had the largest R_ct_ value, followed by the CaCO_3_ and Mg(OH)_2_ mixed layer, and then the Mg(OH)_2_ layer. These results indicate that the CaCO_3_ layer worked better as a protective layer than the others. At the same time, the CPE_1_ values tend to be the opposite of R_ct_. This is because the active area of metal dissolution decreases as the calcareous deposition becomes wider and thicker on the metal surface. The CPE_2_ values of the CaCO_3_ layer were higher than those of the other calcareous deposit layers, meaning that the CaCO_3_ layer was the thickest. This is in agreement with the SEM image results. Figure 8 shows the total resistance values of the bare steel and the three types of calcareous deposits. All specimens with calcareous deposits had a higher total resistance than the bare specimen, demonstrating that the calcareous deposits provided protection to the bare specimens.

### 3.4. Corrosion Acceleration Test

**Figure 9:**
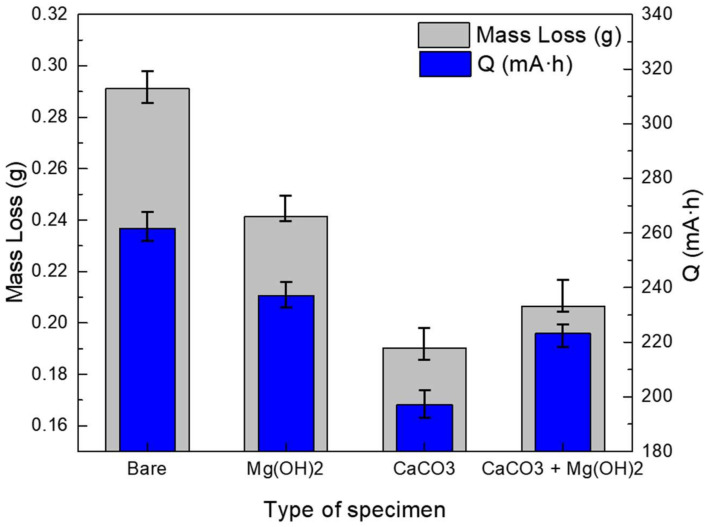
Mass losses of corrosion specimen and quantities of inflow current after potentiostatic acceleration test.

A potentiostatic test was performed at 3.5 V_SCE_ for 100 h to verify the stray current corrosion mitigation of the calcareous deposits [35]. Figure 9 shows the total electric charge at the current inflow area when 3.5 V_SCE_ was applied for 100 h, along with the mass loss of the specimen at the current outflow area. The total electrical charge value was obtained using the following equation [14,36]:

(6) $$Q=\int_{ti}^{tf}I\;dt$$

**Figure 10:**
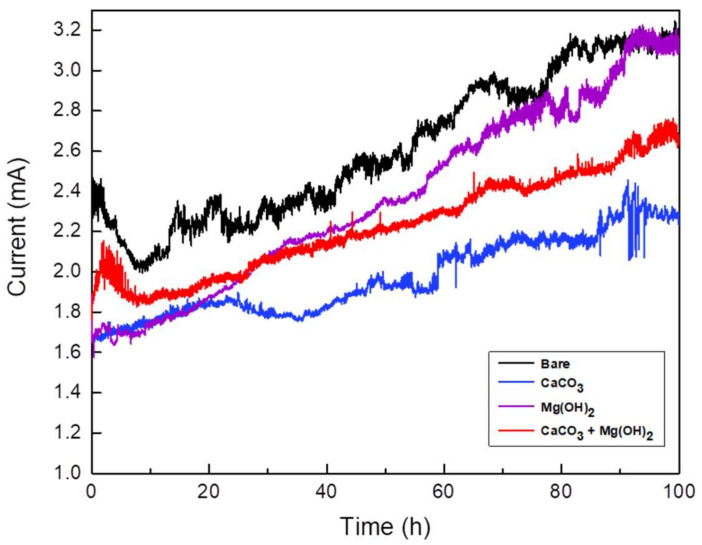
Current vs. time curves during potentiostatic test (applied voltage: 3.5 V_SCE_; solutions: synthetic soil solution; testing time: 100 h).

Since the calcareous deposits decreased the inflow current, the total electric charge values of all specimens with a calcareous deposit were lower than that of the bare specimen. During the corrosion acceleration tests, a crack occurred in the unstable Mg(OH)_2_ layer. Therefore, the total electric charge of the specimen with the Mg(OH)_2_ layer is higher than those of the other specimens with calcareous deposits. In addition, the total electric charge of the specimen with the CaCO_3_ layer is lower than those of the other specimens, meaning that the CaCO_3_ layer was the most protective against inflow current. Figure 10 shows that the specimen on which CaCO_3_ is deposited receives the lowest inflow current and acts as a stable electrical barrier layer. This is because the CaCO_3_ layer grows in a solid and stable form, compared to the Mg(OH)_2_ deposition layer, and without the hindering of Mg(OH)2, it forms a thicker deposition layer than other layers [28,29]. Therefore, it acts as an electrical and physical barrier that blocks the external inflow current more efficiently than other layers. The total electric charge of the specimen with the CaCO_3_ and Mg(OH)_2_ mixed layer is higher than that of the specimen with the CaCO_3_ layer, and lower than that of the specimen with the Mg(OH)_2_ layer. Generally, more current flowing in means more current flowing out. Therefore, as current inflow increases, the areas where the current outflows become more susceptible to corrosion. As a result, among the specimens representing the current outflow area, the mass loss was the lowest in the specimen with the CaCO_3_ layer deposited. The mass reduction then increased in the following order, with respect to the deposit layer composition: CaCO_3_; the CaCO_3_ and Mg(OH)_2_ mixed layer; the Mg(OH)_2_ layer; and the bare specimen.

## 4. Conclusions

This study evaluated the stray current corrosion mitigation of calcareous deposits on carbon steel in a synthetic soil solution using the electrochemical tests, SEM, EDS, and XRD. On the basis of the experiments, the following conclusions can be drawn:

- In the potentiostatic test, the current densities in all types of calcareous deposit layers decreased with the test time;
- The specimen with the CaCO_3_ layer had the lowest current density. In the surface analysis, the specimen in the CaCO_3_ solution has the thickest layer compared to the Mg(OH)_2_ and mixed solutions;
- In the EIS test, the specimen immersed in the CaCO_3_ solution had the highest R_film_ and R_ct_, indicating that the calcareous deposit of CaCO_3_ is the most protective layer;
- The potentiostatic acceleration test demonstrated that the CaCO_3_ layer had the lowest total electric charge among the specimens with calcareous deposits. In addition, the mass loss by the current outflow was the lowest in those with a CaCO_3_ layer.

Consequently, stray current corrosion can be effectively mitigated if CaCO_3_ powders are buried together with the pipelines and deposited when the soil solution and stray current are introduced to the pipelines.

## Figures and Tables

**Table 1:** Chemical composition of SPW 400 (wt.%).

Fe	C	P	S	Si	Mn
Bal	0.130 max.	0.018 max.	0.070 max.	0.240 max.	0.560 max.

**Table 2:** Chemical composition of synthetic soil solution (ppm).

CaCl_2_	MgSO_4_ 7H_2_O	NaHCO_3_	H_2_SO_4_	HNO_3_
133.2	59.0	208.0	48.0	21.8

**Table 3:** The results of the EIS fitting using the circuit.

Type of Deposit	R_s_	CPE_1_		R_film_	CPE_2_		R_ct_
	(Ω·cm^2^)	CPE	Y_0_ (0 < n < 1)	(Ω·cm^2^)	CPE	Y_0_ (0 < n < 1)	(Ω·cm^2^)
Bare	393.7	1.912 × 10^−4^	0.7527	-	-	-	4785
Mg(OH)_2_	511.3	2.955 × 10^−4^	0.8693	523.5	3.893 × 10^−4^	0.7953	6100
CaCO_3_	568.2	5.955 × 10^−4^	0.7615	865.9	1.702 × 10^−4^	0.7231	15,210
CaCO_3_ + Mg(OH)_2_	375.9	2.923 × 10^−4^	0.7533	643.4	1.795 × 10^−4^	0.7959	8872

## Data Availability

Not applicable.
